# An improved farmland fertility algorithm for many-objective optimization problems

**DOI:** 10.1038/s41598-022-06329-x

**Published:** 2022-02-15

**Authors:** Yanjiao Wang, Peng Gao, Ye Chen

**Affiliations:** 1 School of Electrical Engineering, Northeast Electric Power University, Jilin, 132012 China; 2 China Unicom in Changchun City Branch, Changchun, 130000 China

**Keywords:** Information technology, Information theory and computation

## Abstract

Recent studies on many-objective optimization problems (MaOPs) have tended to employ some promising evolutionary algorithms with excellent convergence accuracy and speed. However, difficulties in scalability upon MaOPs including the selection of leaders, etc., are encountered because the most evolutionary algorithms are proposed for single-objective optimization. To further improve the performance of many-objective evolutionary algorithms in solving MaOPs when the number of the objectives increases, this paper proposes a many-objective optimization algorithm based on the improved Farmland Fertility algorithm (MOIFF). In MOIFF, a novel bio-inspired meta heuristic method proposed in 2018, called Farmland Fertility algorithm (FF), is employed to serve as the optimization strategy. In order to handle MaOPs effectively, FF has been tailored from the following aspects. An individual fitness assessment approach based on cumulative ranking value has been proposed to distinguish the quality of each individual; a novel method based on individual cumulative ranking value to constitute and update the global memory and local memory of each individual is proposed, and a hybrid subspace search and full space search method has been designed to update individuals in the stages of soil optimization and soil fusion. In addition, adaptive environmental selection has been proposed. Finally, MOIFF is compared with four state-of-the art many-objective evolutionary algorithms on many test problems with various characteristics, including the DTLZ and WFG test suites. Experimental results demonstrate that the proposed algorithm has competitive convergence and diversity on MaOPs.

## Introduction

Many-objective optimization problems (MaOPs), multi-objective optimization problems (MOPs) with more than three objectives, have been attracting considerable attention in many fields. On the one hand, many real-world applications can be modeled as MaOPs, such as parameter estimation^1,2^, engine calibration^3^, and community detection^4^. On the other hand, MOPs have not been well solved when the number of objectives increases. To overcome this challenge, lots of many-objective evolutionary algorithms which adopted an evolutionary algorithm, have been proposed to improve the performance in solving MaOPs. However, nearly all evolutionary algorithms solve single-objective optimization problems. Many-objective evolutionary algorithms (MaOEAs) must add other many-objective processing approaches, including Pareto-based approaches, decomposition-based approaches, and indicator-based approaches. Many-objective evolutionary algorithms contain two following major parts: the evolutionary algorithm and many-objective processing approach.

In recent years, many promising many-objective evolutionary algorithms have been proposed. Bader et al. proposed the hypervolume (HV) estimation algorithm (HypE)^5^. Yang et al. proposed a grid-based evolutionary algorithm (GrEA)^6^, which introduces the concepts of grid dominance and grid differentiation, and evaluated the relationships among individuals in the grid environment. Yuan et al. proposed a new theta-dominance^7^, in which the right-to-use weight vector divides each solution into a niche and completes the evaluation and selection of the solution in each niche. Zhang et al. proposed a many-objective optimization algorithm based on an improved r-dominance^8^, which adopts the dynamic value strategy of the non-r-dominance threshold with nonlinear reduction based on r-dominance. Cheng et al. proposed a reference vector-guided evolutionary algorithm^9^, which introduces a new aggregation function APD to judge the merits and demerits of the solution by the angle between the solution and the associated weight vector. Zhu et al. presented a new linear weighted minimal/maximal dominance (LWM-dominance) and a new many-objective optimization algorithm based on LWM-dominance^10^. Sun et al. proposed an inverted generational distance (IGD) indicator-based evolutionary algorithm^11^. Dabba et al. applied the artificial fish swarm optimization algorithm to solve the MaOPs^12^. Zhao et al. developed a decomposition-based many-objective artificial bee colony algorithm with reinforcement learning^13^. Wu et al. introduced a novel many-objective Brain Storm optimization algorithm^14^. Zhang et al. proposed a hybrid multi-agent Coordination Optimization Algorithm (MCO)^15^ that, applies a coordination mechanism to accelerate convergence. Guo et al. presented a many-objective optimization with an improved shuffled frog leaping algorithm^16^. Liu et al. proposed a novel multi-objective optimization algorithm based on Bacterial Foraging algorithm^17^. Uzman et al. developed a many-objective hybrid Bacterial Foraging algorithm^18^ and a multi-objective artificial butterfly optimization algorithm^19^. Li et al. proposed a many-objective optimization algorithm based on the R2 indicator and objective space partition^20^. This algorithm, uses a double-layer file correction strategy to provide each solution a different priority when selecting the candidate solutions, in order to give priority to the discrete solutions, and was proved to solve seriously missing diversity. In order to understand the composition of the above representative many-objective evolutionary algorithms more clearly, we summarize these algorithms in Table 1.

**Table 1:** Representative many-objective evolutionary algorithms.

Many-objective processing approaches		Evolution strategy	Many-objective evolutionary algorithm	Number of objectives	References
Pareto-based approaches	Pareto-dominance	GA	NSGAII^21^	2, 3	Deb et al.^21^
		Random Frog Hopping Algorithm	MOSFLA^16^	2, 3, 5	Guo et al.^16^
		Chimpanzee Algorithm	MOBO^22^	2, 3	Amit Kumar Das et al.^22^
	Grid-dominance	GA	GRID^6^	4, 5, 6, 8, 10	Yang et al.^6^
	r-Dominance	PSO	r-MOPSO^8^	3, 5, 10	Zhang et al.^8^
	theta-Dominance	SBX operator and variation	$\theta$-DEA^7^	3, 5, 8, 10, 15	Yuan et al.^7^
	LWM-dominance	GA	LWM-NSGAII^10^	5, 10, 15, 20	Zhu et al.^10^
Decomposition-based approaches	Weighted sum approach, Weighted Tchebycheff approach, Penalty-based boundary intersection	GA	MOEA/D^23^	2, 3, 4	Zhang et al.^23^
	APD Function	GA	RVEA^9^	3, 6, 8, 10	Cheng et al.^9^
	Weighted sum approach	Artificial Fish Swarms Algorithm (AFSA)	MOAFS^12^	5, 6	Ali Dabba et al.^12^
		Butterfly Optimization Algorithm (BOA)	MOABO^19^	2, 3, 10	Rodrigues et al.^19^
	Reference point-based method	Artificial Bee Colony Algorithm (ABC)	MaOABC/D-LA^13^	3, 5, 10, 15	Zhao et al.^13^
		Bat Algorithm (BA)	MaOBAT^18^	2, 3, 5, 7, 10	Perwaiz et al.^18^
	Penalty-based boundary intersection	Brain Storm Optimization Algorithm (BSO)	MaOBSO^14^	5, 10	Wu et al.^14^
	Reference point + Pareto-dominance	Cuckoo Search (CS)	HMaOCS^24^	2, 3, 4, 6, 8, 10	Cui^24^
Indicator-based approaches	HV indicator	GA	HypE^5^	2, 3, 5, 7, 10, 25, 50	Bader et al.^5^
	IGD indicator	GA	MaOEA/IGD^11^	8, 15, 20	Sun et al.^11^
	IGD indicator	collaborative optimization control	MoMCO^15^	3, 5, 8, 10	Zhang et al.^15^
	$\varepsilon +$ indicator	Bacterial Foraging Algorithm	HMOBFA^17^	3, 5, 8	Liu et al.^17^
	R2 indicator	PSO	R2-MOPSO-II^20^	3, 5, 8, 10, 15	Li et al.^20^

The following conclusions could be drawn from the above literature. Compared with Pareto-based approaches and indicator-based approaches, decomposition-based approaches are more widely used in handling MaOPs. In addition, most MaOEAs are proposed by improving many-objective processing approaches or adopting some evolutionary strategies proposed recently with excellent performance. MaOEAs adopting evolutionary strategies with excellent convergence accuracy and speed, such as Bat Evolution Strategy and Cuckoo Strategy, have better convergence and diversity than those with classical evolutionary strategies, such as GA, PSO, and ABC. MaOEAs with some improved many-objective processing approach and a novel excellent evolutionary strategy simultaneously are likely to achieve the promising performance.

To improve the performance of MaOEAs in solving MaOPs when the number of the objectives increases, we proposed a many-objective optimization method based on an improved Farmland Fertility algorithm (MOIFF). Our main innovation and contributions in this paper can be summarized as follows.

1. Farmland Fertility algorithm (FF) as a novel bio-inspired meta heuristic method proposed in 2018, is employed to serve as the optimization strategy of MOIFF. FF performs better than many well-known meta-heuristic methods (including GA, DE, PSO, and ABC) in terms of convergence accuracy, stability, and speed FF. An improved FF algorithm (IFF) has been proposed in 2020^25^. However, FF and IFF are designed for solving complex single-objective optimization problems. In order to handle MaOPs effectively, FF has been tailored from the following aspects in this study: First, we propose a novel individual fitness assessment approach based on the cumulative ranking value to distinguish the advantages and disadvantages of each individual in MaOPs. Second, according to the characteristics of MaOPs, we proposed a novel method based on individual cumulative ranking value to constitute and update the global memory and local memory of each individual, and propose a hybrid search mode combining subspace search and full space search to update individuals at the stages of soil optimization and soil fusion.
2. The experimental results have proved the dual aggregation functions-based environmental selection is a representative and promising many-objective processing approach. However, satisfactory diversity is hard to obtain, since offspring individuals are selected randomly and some holes may appear in Pareto front (PF). We propose a novel adaptive environmental selection method to address these issues. It not only avoids the blindness of random selection but also satisfies the requirements of convergence and diversity of MaOEAs at different stages of algorithm evolution.

Finally, the proposed MOIFF is compared with four state-of-the art many-objective evolutionary algorithms on many test problems with various characteristics, including DTLZ and WFG test suites. Experimental results demonstrate that the proposed algorithm has competitive convergence and diversity on MaOPs.

The rest structure of the paper is organized as follows: “Methods” introduces the relevant theoretical knowledge, including the basic principles of the original FF and a representative many-objective processing approach; “Proposed method” describes the innovation, the principle, procedures, and detailed operations of our proposed MOIFF; “Results and discussion” compares the performance of MOIFF against four state-of-the art many-objective evolutionary algorithms; “Conclusions” concludes the full text and points out the issues to be studied in future.

## Methods

### Farmland fertility algorithm

In the real world, farmers apply different fertilizers to farmlands with different soil qualities. By simulating the above behavior, Farmland Fertility algorithm (FF) has been proposed to handle single-objective optimization problems. In FF, the fertilization schemes and soil qualities of farmlands are equivalent to individuals and their fitness values, respectively. The section of the farmland with the worst soil quality selected the best fertilization scheme, and for the other sections of the farmland, the fertilization schemes are randomly selected. Finally, the soil quality of farmland can be effectively improved through continuous improvements in fertilization schemes. The pseudo-code of FF is shown in Algorithm 1.
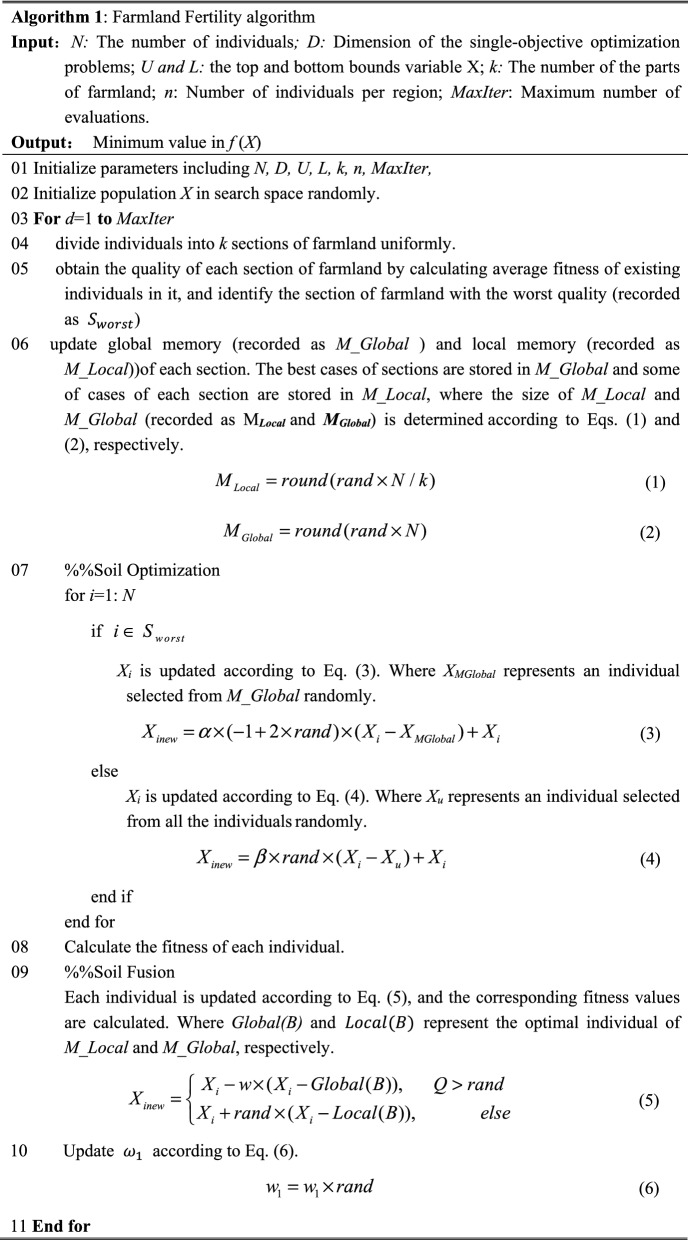


### A representative many-objective processing approach

In 2019, Zhang et al. proposed a novel many-objective processing approach in decomposition-based coevolutionary algorithm (DECAL), named dual aggregation functions-based environmental selection, which consists of two following aggregation functions with complementary strengths: the volume (VOL) function and the KNEE function. Where VOL function is strong at facilitating the convergence of individuals, while KNEE function is designed to maintain the diversity of the population. Unlike the most commonly used PBI approach, the VOL function and the KNEE function proposed in DECAL are parameter-free, and more importantly, obtain better convergence and diversity in MaOPs. For the above reasons, we adopt the dual aggregation functions-based environmental selection proposed in DECAL as the many-objective processing approach of our MOIFF. The pseudo-code of DECAL is shown in Algorithm 2.
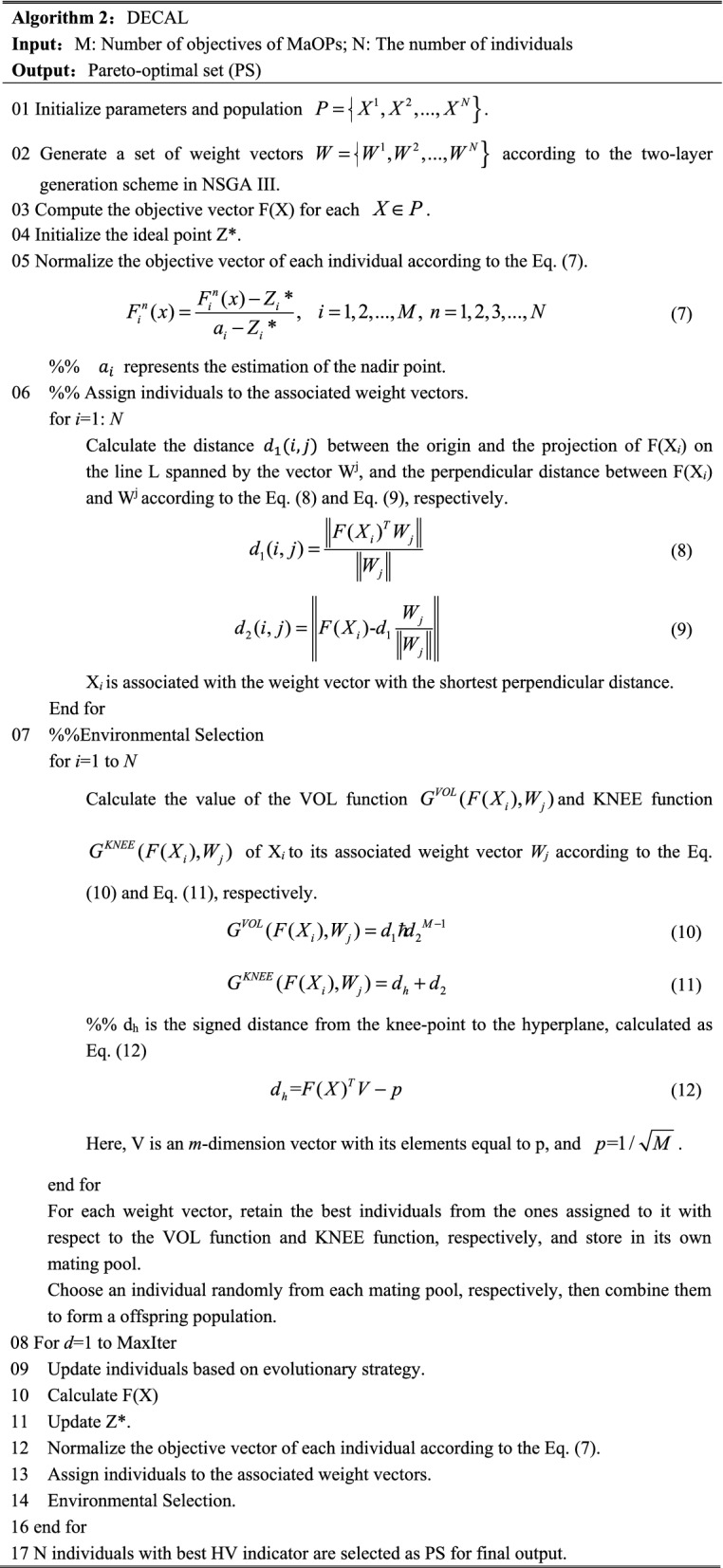


## Proposed method

We proposed a many-objective optimization algorithm based on an improved Farmland Fertility algorithm (MOIFF) to improve the convergence and diversity of MaEAs. FF is employed to serve as the main evolutionary strategy of MOIFF, and dual aggregation functions-based environmental selection with the VOL and KNEE functions is improved as the multi-objective processing approach of MOIFF. The basic procedure of the proposed MOIFF is similar to those of most decomposition-based MaEAs. The pseudo-code of MOIFF is shown in Algorithm 3.
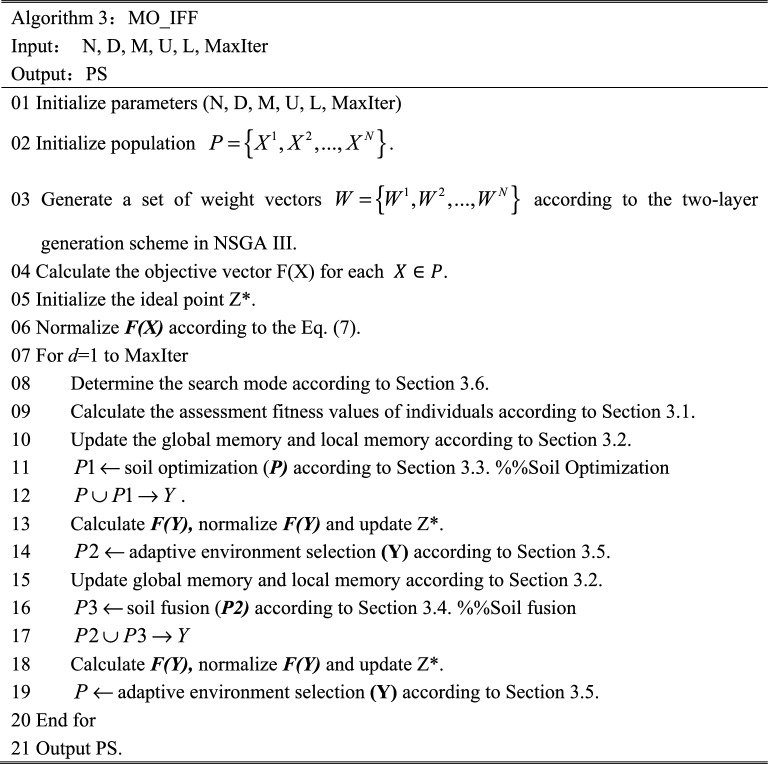


### Novel individual fitness assessment approach based on cumulative ranking value

To evaluate effectively the quality of individuals in MaOPs, we proposed a novel individual fitness assessment approach based on cumulative ranking value. The main motivation is as follows. For decomposition-based MaEAs, individuals associated with the same weight vector can be compared in performance. Suppose that all the individuals in MaOPs are associated with the same weight, then, they can be compared. However, decomposition-based approaches commonly contain some evenly spread weight vectors, and almost all individuals are associated with different weight vectors. The performance of each individual should be evaluated by all weight vectors instead of one. In addition, at the beginning of iteration, MaEAs should facilitate the convergence of individuals; at the end of iteration, attention should be shifted to diversity.

Basing from the above motivation, we proposed the following method to evaluate the quality of each individual for MaOPs. Step 1: At the beginning of the iteration, for each individual, calculate the VOL function value on each weight vector by using to Eq. (10); at the end of iteration, calculate the KNEE function value by using Eq. (11). Step 2: Sort the individuals associated with the same weight vector with respect to the VOL function or the KNEE function. Thus, each individual will be assigned to *N* ranking values, where *N* is equal to the size of a set of weight vectors. Step 3: For each individual, accumulate all ranking values as its novel assessment fitness value, recorded as *s_sort(i)*. Obviously, the smaller the cumulative sorting value *s_sort(i)* of an individual, the better the individual.

**Figure 1:**
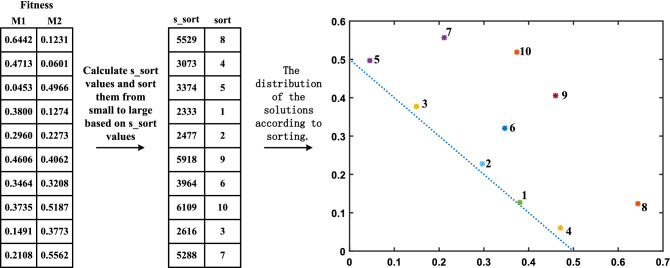
Principle and effect of the novel individual fitness assessment approach based on cumulative ranking value.

Figure 1 displays an example of handling two-objective DTLZ1 to further illustrate the effectiveness of the above fitness assessment approach based on cumulative ranking value. The final distribution of the solutions shows that the individuals with the first five cumulative ranking values are distributed close to the true Pareto front (PF); the larger the cumulative ranking value of the individual is, the father away from the true PF. The above finding shows that our proposed novel individual fitness assessment approach based on the cumulative ranking value is effective.

### Mechanism of updating global memory and local memory

The decomposition-based MaEAs decompose a many-objective optimization problem into a number of single-objective sub-problems by an even set of weight vectors. Each sub-problem is defined by a weight vector, and if weight vectors are adjacent to each other, the optimal solutions of the sub-problems associated with them are also very close. Therefore, with the extension of evolution, the individuals associated with neighborhood weight vectors are similar to some extent. Obviously, each individual and its neighboring individuals form a region naturally; thus, dividing each individual into regions like FF is not necessary. However, a new mechanism must be proposed to compose and update local memory and global memory in MaOPs. Basing from the above analysis, we proposed the following mechanism based on individual cumulative sorting values to compose and update local memory and global memory.

**Figure 2:**
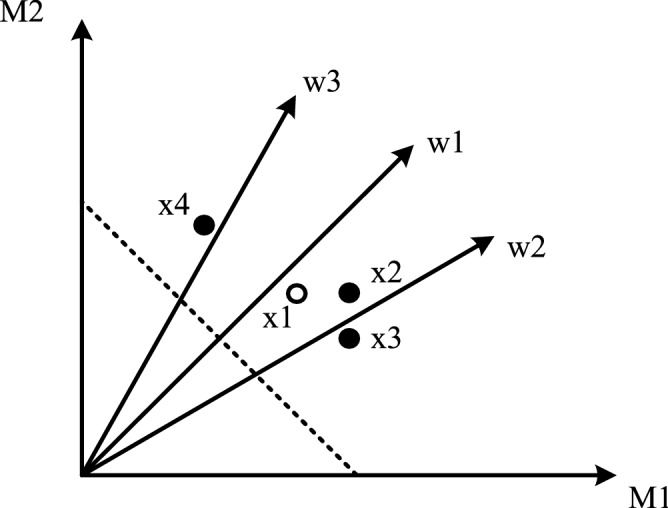
Schematic of the neighbor population.

1. Mechanism of composing and updating global memory
  At each iteration, *M*_*Global*_ individuals with the smallest *s_sort* value in the current population are selected and stored them in the global memory directly.
2. Mechanism of composing and updating local memory
  Unlike FF, each local memory is assigned to each individual rather than each region in MOIFF. That is, the number of local memory is equal to the number of individuals. The local memory of each individual is updated with the help of its neighbor population (shown in Fig. 2) as follows. First, for each individual ***X***_***i***_, the weight vector associated with it is determined, and then the T weight vectors closest to the associated weight vector are selected as the neighbor weight vectors. Second, individuals associated with each neighbor weight vector are identified to form the neighbor population of ***X***_***i***_. Finally, *M*_*Local*_ individuals with the smaller cumulative sorting values in the neighbor population of ***X***_***i***_ are selected as the local memory of ***X***_***i***_ directly.

In Fig. 2, dotted lines represent the true PF; w1, w2, and w3 represent the different weight vectors; and x1, x2, x3, and x4 represent four individuals in the current population, where x1 is associated with vector w1, x2 and x3 with weight vector w2, and x4 with weight vector w3. For the individual x1, w2 and w3 are the neighbor weight vectors of the associated weight vector w1 with it. Individuals x2 and x3 are associated with weight vector w2, and x4 is associated with weight vector w3. Individuals x2, x3, and x4 compose the neighbor population of individual x1.

### New individual-updating mechanism for soil optimization

To handle MaOPs, we propose a novel individual-updating mechanism for soil optimization in view of the characteristics of decomposition-based MaEAs, described as Eq. (13). The main motivations and ideas are as follows: to facilitate the convergence and promote the diversity of MOIFF, individuals with the poorer convergence should learn from the excellent individuals in the neighborhood, and the others should explore new locations by further communication with other individuals different from itself.

13 $$X_{{inew}} = \left\{ {\begin{array}{*{20}l} {\alpha \times ( - 1 + 2 \times rand) \times (X_{i} - X_{{i\_ML}} (best)) + X_{i} ,} \hfill & {s\_sort(X_{i} ) \in N/k} \hfill \\ {\beta \times rand \times (X_{i} - X_{{i\_ML}} (others)) + X_{i} ,} \hfill & {else,} \hfill \\ \end{array} } \right.$$

where *X*_*i_ML*_*(best)* and *X*_*i_ML*_*(others)* represent the best individual and another random individual besides the best individual in ***X***_***i***_’s local memory, respectively; and *k* is a constant, generally, *k* = 4.

The above individual-updating mechanism proposed for soil optimization has the following advantages. On the one hand, the *N/k* individuals with poor convergence and diversity learn from the excellent individuals in their local memory randomly, which provides the direction of evolution within the neighborhood and maintains the diversity of the whole population. On the other hand, others learn from other random individual besides themself, which favors exploration of new locations, and can make some weight vectors without associated individuals may be associated with some individuals. Therefore, the diversity of MOIFF will be improved.

### A new individual-updating mechanism for soil fusion

As shown in Eq. (5), in the soil fusion stage of FF, all individuals only learn from the best individual in the global memory or local memory, which improves the convergence speed of FF to a certain extent. Unfortunately, it is unsuitable for decomposition-based MaEAs. Therefore, we propose a new individual-updating method for soil fusion, described as Eqs. (14) and (15). The main motivations and ideas are as follows: in the soil fusion stage, only the individual in the global memory and the best individual in each local memory are exploited many times rather than all the individuals, and minor perturbations are made around them. However, considering that the number of these individuals is small, i.e., the evolutionary information is very limited, and each individual has carried some evolutionary information related to the convergence of corresponding sub-problems during iterations. Therefore, each excellent individual should be updated by the minor perturbations around themselves and add some evolutionary information of other individuals.

14 $$X_{{inew}} = \left\{ {\begin{array}{*{20}l} {X_{{MG}} (random) \times randn,} \hfill & {Q > rand} \hfill \\ {X_{{i\_ML}} (best) \times randn,} \hfill & {else,} \hfill \\ \end{array} } \right.$$

15 $$X_{{inew,j}} = \left\{ {\begin{array}{*{20}l} {X_{{inew,j}} ,} \hfill & {CR > rand} \hfill \\ {X_{{i,j}} ,} \hfill & {else,} \hfill \\ \end{array} } \right.$$

where, $X_{MG} (random)$ represents a random individual selected from global memory, $X_{i\_ML} (best)$ represents the best individual stored in X_i_'s local memory, CR is the crossover probability, and generally, $CR \in [0.6,0.8]$.

### Adaptive environmental selection

As described in Algorithm 2, dual aggregation functions-based environmental selection in DECAL consists of the two following steps: Step 1, for each weight vector, select the individual with the best VOL function value and the individual with the best KNEE function value from all the associated individuals with it respectively; Step 2, for each weight vector, randomly select one from the individuals identified in step 1 as an individual of the offspring population. Obviously, some weight vectors may not be associated with any individual. Therefore, the number of individuals in the offspring population may be smaller than the initial size of individuals, and satisfactory diversity is hard to obtain because some holes may appear in PF. In addition, the random selection in step 2 has a certain blindness, which does not satisfy the requirements of convergence and diversity of many-objective optimization algorithms at different stages of algorithm evolution.

To address these above issues, this article proposes a new adaptive environment selection, as shown in Algorithm 4.
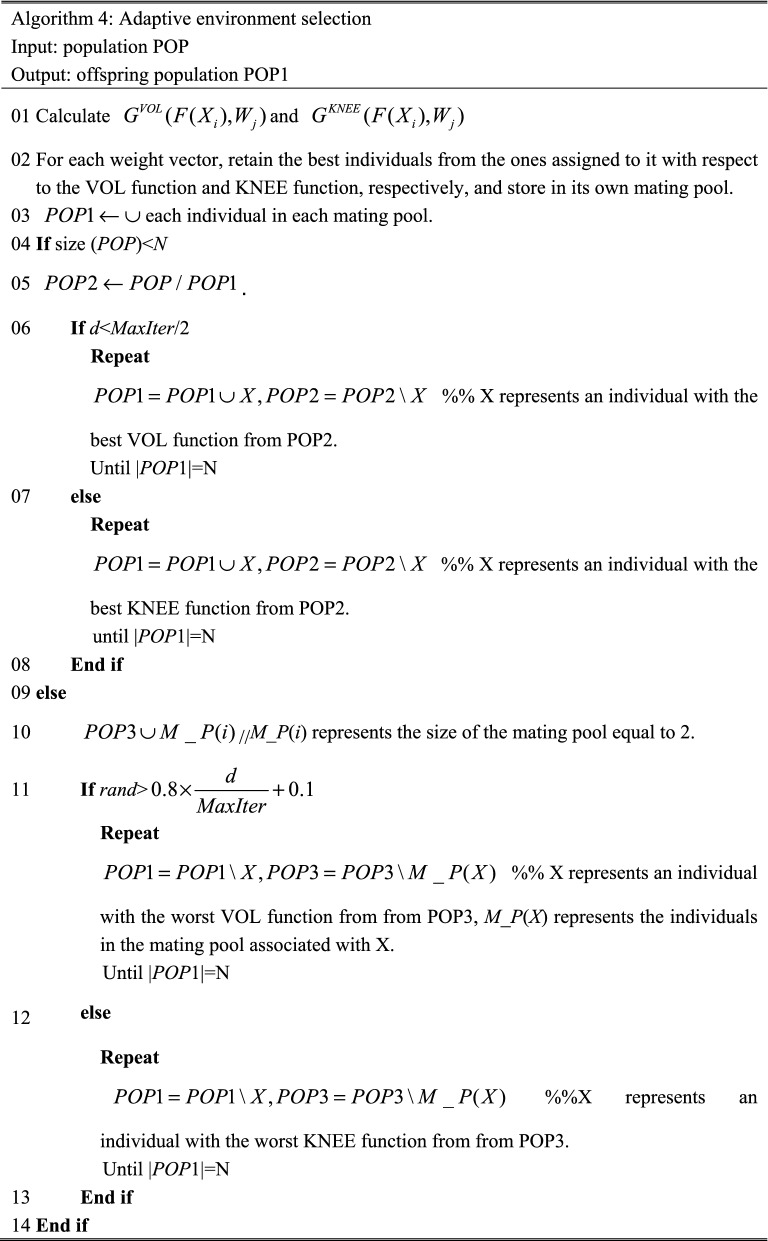


As shown in Algorithm 4, the adaptive environment selection proposed in this paper has the following advantages: First, when the size of the offspring population is less than the initial setting number of weight vectors, we will select some individuals into the offspring population, and vice versa. Finally, the size of the offspring population obtained by adaptive environment selection is equal to the initial setting number of weight vectors, which further guarantees that the PF is even distributed; Second, at the begging of iterations, the offspring population obtained by adaptive environment selection contains many individuals with better VOL fitness. At the end of iterations, the offspring population contains many individuals with better KNEE fitness. It not only avoids the blindness of random selection but also satisfies the requirements of convergence and diversity of many-objective optimization algorithms at different stages of algorithm evolution.

### Hybrid subspace-full space search mode

For MaOPs, at the begging of iteration, the set of all the solutions is commonly far from PS. If each individual performs a complete search in the D-dimensional search space, called the full-space search mode, then the individual can obtain some promising evolutionary directions from such huge searching space. As the iteration progresses, the individuals are gradually close to the true PS. At this moment, some individuals have achieved the global optimum in most dimensions, whereas only a few dimensions are far from the global optimum. In this case, if individuals still adopt the full-space search mode of FF to evolve, the good dimensions may change greatly, which may cause the population to deviate from the excellent evolution directions, and the convergence of algorithm may be slow or fail to obtain the true PF. The current individuals are better suited for small-scale searches. Thus, the above-mentioned issues can be easily addressed, if individuals do not adopt the full-space search mode but only search on a dimension, called subspace search. In summary, the full-space search mode refers to all dimensions of an individual being updated in accordance with the established search strategy in the stages of soil optimization and soil fusion, and the subspace search mode means that during the stages of soil optimization and soil fusion, only one dimension of an individual is updated in accordance with the established search strategy, while the other dimensions are unchanged.

The subspace search mode updates only one dimension of individual at one iteration. Thus, if individuals adopt the subspace search mode to update themselves for many iterations, the convergence of the algorithm may be slow down instead. Therefore, when the subspace search mode does not obtain a better PF, the full space search mode should be used again for further search. That is, the full space search mode and subspace search mode should alternate. Basing from the above ideas, we propose a hybrid subspace-full space search mode. The conversion condition between the subspace and full-space search mode are as follows.

#### Conversion condition between subspace and full-space search modes

As shown in “Novel individual fitness assessment approach based on cumulative ranking value”, for each weight vector, the associated sub-problem is solved better, as the smallest *s_sort* associated with it (denoted as *pbest*) is smaller. As the iteration progresses, the change in *pbest* can be used to judge whether the evolution slow down, fail to update the individual into a better one, and fall into a local optimum. Considering the above, we take the change in *pbest* as the conversion condition between the subspace search mode and full-space search mode as follows: First, initialize parameters including *c* = *0*, *c*1, and *c*2, where *c*1 is used to determine whether *pbest* has changed before and after the iteration, and *c*2 is used to determine the number of iterations that *pbest* remains unchanged. Second, for each weight vector, calculate the Euclidean distance ***Dis(i)*** between ***pbest***_***i***_ obtained at this iteration and the last iteration and then calculate the mean (denoted as *average_Dis*) of all ***Dis(i)***, as shown in Eq. (16). If *average_Dis* is less than *c*1, then c = c + 1; otherwise, c = 0. When *c* is equal to *c2*, the search mode is converted, i.e., the full space search mode is converted to the sub-space search mode, and vice versa.

16 $$average_{Dis} = mean\left( {\sum\limits_{i = 1}^{N} {\sqrt {\sum\limits_{j = 1}^{D} {(pbest_{i}^{d} (j) - pbest_{i}^{d - 1} (j))^{2} } } } } \right),$$

where, $pbest_{i}^{d} (j)$ represents the ***j*-**th dimension of the smallest *s_sort* associated with the *i*-th weight vector (*W*_*i*_) at the *d*-th iteration.

#### Description of subspace search mode

When the subspace search mode is adopted, if the updated dimension is selected in accordance with the order of dimensions, it can better guarantee that each dimension can search more finely during the limited iterations compared with the updated dimension selected randomly. Therefore, in our proposed subspace search mode, when converting to the subspace search mode, the updated dimension of each individual is selected in accordance with the order of dimensions at each iteration, which is similar to the method proposed in^25^, detailed as follows. At the last iteration, dimension *d* is selected to update, and at this iteration, dimension *d* + *1* is selected to update. When all the dimensions have been updated by adopting the subspace search mode, unlike the method proposed in^25^, even if they fail to meet the conversion condition between the subspace and full-space search modes, all individuals adopt the full-space search mode in the next iteration.

Suppose that the *flag*-th dimension needs to be updated, the subspace search modes for soil optimization and soil fusion are as follows.

The subspace search mode for soil optimization and soil fusion is shown in Eq. (17).

17 $$X_{inew,flag} = \left\{ {\begin{array}{*{20}l} {\alpha \times ( - 1 + 2 \times rand) \times (X_{i,flag} - X_{i\_ML,flag} (best)) + X_{i,flag} ,{\kern 1pt} {\kern 1pt} {\kern 1pt} {\kern 1pt} {\kern 1pt} {\kern 1pt} {\kern 1pt} s\_sort(X_{i} ) \in {N \mathord{\left/ {\vphantom {N k}} \right. \kern-\nulldelimiterspace} k}} \\ {\beta \times rand \times (X_{i,flag} - X_{i\_ML,flag} (others)) + X_{i,flag} ,{\kern 1pt} {\kern 1pt} {\kern 1pt} {\kern 1pt} {\kern 1pt} {\kern 1pt} {\kern 1pt} {\kern 1pt} {\kern 1pt} {\kern 1pt} {\kern 1pt} {\kern 1pt} {\kern 1pt} {\kern 1pt} {\kern 1pt} {\kern 1pt} {\kern 1pt} {\kern 1pt} {\kern 1pt} {\kern 1pt} {\kern 1pt} {\kern 1pt} {\kern 1pt} {\kern 1pt} {\kern 1pt} {\kern 1pt} {\kern 1pt} {\kern 1pt} {\kern 1pt} {\kern 1pt} {\kern 1pt} {\kern 1pt} {\kern 1pt} {\kern 1pt} {\kern 1pt} {\kern 1pt} {\kern 1pt} {\kern 1pt} {\kern 1pt} {\kern 1pt} {\kern 1pt} {\kern 1pt} {\kern 1pt} {\kern 1pt} {\kern 1pt} else.{\kern 1pt} } \\ \end{array} } \right.$$

At the stage of soil fusion, to guarantee that the new offspring individuals are different from themselves and facilitate convergence, each individual performs the crossover operation between the disturbed individual and the disturbed selected excellent individual, as shown in Eq. (18).

18 $$X_{inew,flag} = \left\{ {\begin{array}{*{20}l} {X_{MG,flag} (random) \times randn,{\kern 1pt} {\kern 1pt} {\kern 1pt} {\kern 1pt} {\kern 1pt} {\kern 1pt} {\kern 1pt} Q > rand} \\ {X_{i\_ML,flag} (best) \times randn,{\kern 1pt} {\kern 1pt} {\kern 1pt} {\kern 1pt} {\kern 1pt} {\kern 1pt} {\kern 1pt} {\kern 1pt} {\kern 1pt} {\kern 1pt} {\kern 1pt} {\kern 1pt} {\kern 1pt} {\kern 1pt} {\kern 1pt} else.{\kern 1pt} } \\ \end{array} } \right.$$

### Complexity analysis

Given an MaOP with *M* objectives in a *D*-dimensional decision space. Assume that the population size of each swarm is NP. the time complexity of our proposed MOIFF is dominated by the operators in the for loop (lines 08–19 in Algorithm 3). In each iteration, determining the search mode (line 08) requires $O(D \times NP)$ time. Calculating he assessment fitness values of individuals (line 09) costs $O(NP{ + }M \times NP^{2} )$ time. Updating the global memory and local memory (line 10) takes $O(M_{Global} { + }M_{Local} )$ time, where *M*_*Global*_* and* $M_{Local}$ represent the size of the global memory and the local memory. The soil optimization component (line 11) requires $O(D \times NP)$ time. The component of line 12 costs $O(M \times NP^{2} )$ time. Adaptive environment selection (line 14) requires $O(NP^{2} )$ time. The soil fusion takes $O(D \times NP)$ time. Therefor, the overall time complexity of our proposed MOIFF is $O(M \times NP^{2} )$.

## Results and discussion

### Test problems

In the empirical studies, two well-known test suites for many objective optimizations, the DTLZ test suite^26^ and the WFG test suite^27^ are chosen. To obtain a full comparison, all test instances in DTLZ and WFG are considered in this paper. All these test problems are scalable to any number of objectives, where $M \in \left\{ {3,5,8,10,15} \right\}$ in this paper. According to^26^, the number of decision variables V = M + k − 1 for the DTLZ test suit, where M represents the number of objectives, k = 5 for DTLZ1, and k = 10 for DTLZ2-7. For all WFG test problems, the number of decision variables is set as V = r + l, where the position-related variable r = 2 × (M − 1) and the distance-related variable l = 20, as suggested in^27^.

### Algorithm and parameter

In order to verify the validity of MOIFF proposed in this paper, we consider four state-of-the-art many-objective evolutionary algorithms, including NSGA III^28^, MOEA/D^23^, RVEA^9^, and ARMOEA^29^. To ensure the fairness of the comparison, the population size and the termination condition of each algorithm are consistent on the same test instance. The population size *N* is equal to the size of the reference vectors, which are used for different numbers of objectives and are summarized in Table 2, where *H*_1_ and *H*_2_ are the number of divisions of the boundary layer and inside layer, respectively. The termination condition of each algorithm is the maximum number of fitness estimations (MFE), summarized in Table 3. In addition, other parameters used in each algorithm are summarized from the original literature, as shown in Table 4.

**Table 2:** Population size setting.

Number of objectives *M*	Population size *N*
3	91 (*H* = 12)
5	210 (*H* = 6)
8	156 (*H*_1_ = 3, *H*_2_ = 2)
10	275 (*H*_1_ = 3, *H*_2_ = 2)
15	135 (*H*_1_ = 2, *H*_2_ = 1)

**Table 3:** Maximum number of fitness estimations for different test problems.

Problem	*M* = 3	*M* = 5	*M* = 8	*M* = 10	*M* = 15
DTLZ1	36,400	126,000	117,000	275,000	202,500
DTLZ2	22,750	73,500	78,000	206,250	135,000
DTLZ3	91,000	210,000	156,000	412,500	270,000
DTLZ4	54,600	210,000	195,000	550,000	405,000
DTLZ5	54,600	210,000	187,200	168,000	270,000
DTLZ6	36,800	73,500	117,000	224,000	270,000
DTLZ7	54,600	210,000	195,000	550,000	405,000
WFG1-WFG9	92,000	212,100	156,000	276,100	136,000

**Table 4:** Setting of parameters in each algorithms.

MaOEAs	Parameters
NSGAIII	*P*_*c*_ = 1.0, *P*_*m*_ = 1/*V*, ${\eta }_{c}=20$, ${\eta }_{m}=20$
MOEA/D	*T* = 20, $\delta =0.9$
RVEA	$\alpha =2$, *f*_*r*_ = 0.1
MOIFF	*T* = *N*/4, *CR* = 0.8, $\alpha =0.6$, $\beta =0.4$

### Results on DTLZ test suite

In this section, we conduct three experiments to verify the validity of MOIFF proposed in this paper on the DTLZ test suite. The first one is verification of the effectiveness of MOIFF in convergence accuracy. The second one is verification of the effectiveness of MOIFF in convergence speed. The third one is verification of the effectiveness of MOIFF in convergence stability.

1. Verification of the effectiveness of MOIFF in convergence accuracy

In our empirical studies, IGD and HV are employed to evaluate the performance of each algorithm simultaneously. Each algorithm runs 30 times independently on each test instance to avoid the unfavorable effect of the algorithm evaluation caused by the randomness of a single operation. Tables 5 and 6 show the average and standard deviation of the IGD and HV values over 30 independent runs for the five compared MaOEAs, respectively, where the best average among the five compared MaOEAs is highlighted in bold. In addition, to test the differences for statistical significance, the Wilcoxon rank sum test with a 5% significance level is performed between MOIFF and each of the compared algorithms over each test instance. Symbols “+”, “−” and “=” indicate that the compared algorithm performs significantly better than, worse than, and equivalent to MOIFF in the corresponding column, respectively. The Friedman rank-sum test is performed on the data of Tables 5 and 6 to analyze the overall average performance of these above algorithms. The results are shown in Table 7, where “*avg.rank”* represents the average rank of each algorithm, “*rank”* is the overall rank of five algorithms in average performance.

**Table 5:** Average and standard deviation of the IGD values obtained by the five algorithms on the DTLZ test suite with different numbers of objectives.

Text suite	*M*	NSGAIII	MOEA/D	RVEA	ARMOEA	MOIFF
DTLZ1	3	2.07E−02 (2.99E−04) +	2.10E−02 (4.73E−04) +	**2.07E−02 (1.86E−04) + **	2.08E−02 (2.27E−04) +	3.08E−02 (6.00E−02)
	5	5.27E−02 (4.60E−05)−	5.28E−02 (8.10E−05)−	5.27E−02 (2.80E−05)−	5.27E−02 (5.62E−05)−	**2.42E−02 (4.39E−02)**
	8	1.06E−01 (2.94E−02)−	9.63E−02 (3.28E−04)−	9.71E−02 (2.71E−04)-	9.90E−02 (5.84E−04)−	**2.45E−02 (2.10E−02)**
	10	1.15E−01 (2.37E−02)−	1.09E−01 (1.85E−04)−	1.08E−01 (2.90E−04)−	1.12E−01 (1.21E−03)−	**9.14E−02 (4.82E−02)**
	15	2.04E−01 (2.37E−02) +	**1.64E−01 (7.84E−03) + **	1.77E−01 (5.60E−03) +	1.70E−01 (1.54E−02)−	2.49E−01 (1.13E−02)
DTLZ2	3	5.45E−02 (1.45E−05)−	5.45E−02 (3.84E−06)−	5.46E−02 (1.60E−04)−	5.46E−02 (7.97E−05)−	**2.43E−02 (9.89E−03)**
	5	1.65E−01 (2.72E−05)−	1.65E−01 (9.43E−06)−	1.65E−01 (2.28E−05)−	1.65E−01 (8.57E−05)−	**4.60E−02 (8.90E−03)**
	8	3.37E−01 (6.22E−02)−	3.15E−01 (3.34E−05)−	3.15E−01 (1.41E−04)−	3.26E−01 (1.90E−03)−	**9.83E−02 (9.74E−02)**
	10	4.93E−01 (8.03E−02)−	4.22E−01 (1.69E−04)−	4.20E−01 (4.72E−04)−	4.27E−01 (2.47E−03)−	**1.59E−01 (1.16E−01)**
	15	7.50E−01 (5.07E−02)−	6.23E−01 (7.47E−04)−	6.24E−01 (4.18E−03)−	6.11E−01 (3.01E−03)−	**1.39E−01 (1.40E−01)**
DTLZ3	3	**5.48E−02 (3.04E−04) + **	5.49E−02 (6.97E−04) +	5.48E−02 (3.88E−04) +	5.49E−02 (4.29E−04) +	1.19E−01 (2.50E−01)
	5	1.65E−01 (3.05E−04)−	1.65E−01 (2.15E−04)−	1.65E−01 (7.46E−05)−	1.66E−01 (3.26E−03)−	**7.27E−02 (3.70E−02)**
	8	5.51E−01 (5.16E−01)−	3.89E−01 (1.88E−01) =	3.16E−01 (4.96E−04) =	3.45E−01 (7.68E−03) =	**2.83E−01 (1.84E−01)**
	10	6.50E−01 (6.61E−01)−	4.22E−01 (1.06E−03)−	4.20E−01 (7.19E−04)−	4.29E−01 (4.27E−03)−	**2.25E−01 (1.79E−01)**
	15	1.99E + 01 (1.42E + 01)−	9.40E−01 (2.98E−01)−	6.23E−01 (1.26E−03)−	6.14E−01 (8.73E−03)−	**5.12E−01 (9.88E−02)**
DTLZ4	3	2.79E−01 (2.66E−01)−	3.55E−01 (3.25E−01)−	7.07E−02 (8.75E−02) =	2.60E−01 (2.86E−01)−	**2.19E−01 (2.57E−01)**
	5	1.73E−01 (4.37E−02)−	3.18E−01 (1.58E−01)−	1.65E−01 (6.97E−06)−	1.73E−01 (4.20E−02)−	**1.62E−01 (2.07E−01)**
	8	3.74E−01 (1.00E−01) =	4.82E−01 (9.67E−02)−	3.23E−01 (2.80E−02) =	3.23E−01 (1.52E−03) =	**2.94E−01 (1.76E−01)**
	10	4.38E−01 (4.80E−02)−	5.19E−01 (6.77E−02)−	4.21E−01 (4.41E−04)−	4.27E−01 (2.48E−03)−	**1.62E−01 (1.40E−01)**
	15	7.31E−01 (5.09E−02)−	6.84E−01 (3.13E−02)−	6.25E−01 (1.63E−03)−	6.07E−01 (2.42E−03)−	**3.21E**−**01** **(1.11E**−**01)**
DTLZ5	3	**1.24E**−**02** **(1.56E**−**03) + **	3.39E−02 (1.83E−05)−	7.03E−02 (8.02E−03)−	5.42E−03 (1.25E−04) +	2.96E−02 (4.26E−03)
	5	9.03E−02 (3.22E−02) =	**2.27E**−**02** **(4.54E**−**06) + **	1.99E−01 (1.90E−02)−	5.50E−02 (7.22E−03) +	8.29E−02 (1.94E−02)
	8	3.59E−01 (9.03E−02)−	**2.59E**−**02** **(6.37E**−**06) + **	3.60E−01 (4.04E−02)−	1.04E−01 (2.51E−02) +	1.22E−01 (2.69E−02)
	10	5.02E−01 (1.27E−01)−	**1.99E**−**02** **(1.02E**−**05) + **	3.08E−01 (3.64E−02)−	9.14E−02 (1.39E−02) +	1.49E−01 (3.10E−02)
	15	2.51E−01 (5.64E−02)−	**9.57E**−**02** **(5.05E**−**06) + **	3.06E−01 (9.70E−02)−	1.12E−01 (1.98E−02) +	2.16E−01 (5.19E−02)
DTLZ6	3	2.00E−02 (2.70E−03) +	3.39E−02 (2.04E−05) +	8.53E−02 (1.60E−02)−	**5.00E**−**03** **(6.98E**−**05) + **	4.74E−02 (9.10E−03)
	5	1.66E−01 (7.91E−02)−	**2.26E**−**02** **(1.71E**−**04) + **	1.50E−01 (2.38E−02)−	7.12E−02 (2.11E−02) +	7.78E−02 (1.19E−02)
	8	6.59E−01 (3.43E−01)−	**2.53E**−**02** **(5.79E**−**04) + **	2.92E−01 (6.01E−02)−	1.07E−01 (2.23E−02) +	1.43E−01 (1.86E−02)
	10	2.66E + 00 (1.15E + 00)−	**1.90E**−**02** **(4.56E**−**04) + **	2.40E−01 (5.44E−02)−	1.03E−01 (2.37E−02) +	1.42E−01 (1.90E−02)
	15	4.90E + 00 (1.15E + 00)−	**9.57E**−**02** **(2.53E**−**06) + **	1.87E−01 (1.18E−02)−	1.04E−01 (2.35E−02) +	1.82E−01 (7.98E−02)
DTLZ7	3	**9.79E**−**02** **(8.05E**−**02) + **	1.51E−01 (4.39E−03) =	1.07E−01 (2.76E−03) =	3.06E−01 (2.33E−01) =	2.80E−01 (2.66E−01)
	5	2.79E−01 (1.02E−02) +	5.76E−01 (1.72E−01)−	5.02E−01 (7.27E−03)−	**2.60E**−**01** **(4.14E**−**03) + **	3.46E−01 (3.90E−02)
	8	**7.81E**−**01** **(2.26E**−**02) + **	1.86E + 00 (2.16E−01)−	1.89E + 00 (9.58E−02)−	1.00E + 00 (5.71E−02) =	9.86E−01 (1.17E−01)
	10	**9.68E**−**01** **(7.45E**−**02) + **	3.01E + 00 (3.61E−01)−	2.67E + 00 (1.71E−01)−	1.49E + 00 (1.43E−01) +	2.11E + 00 (3.86E−01)
	15	7.34E + 00 (1.05E + 00)−	**2.77E + 00** **(2.93E**−**01) = **	6.07E + 00 (8.95E−01)−	5.03E + 00 (9.02E−01)−	4.59E + 00 (2.58E + 00)
+/=/−		9/2/24	12/3/20	3/4/28	14/4/17

**Table 6:** Average and standard deviation of the HV values obtained by the five algorithms on the DTLZ test suite with different numbers of objectives.

Text Suite	*M*	NSGA-III	MOEA/D	RVEA	ARMOEA	MOIFF
DTLZ1	3	8.40E−01 (1.85E−03)−	8.38E−01 (2.55E−03)−	8.40E−01 (1.51E−03)−	8.40E−01 (1.57E−03)−	**9.60E**−**01** **(3.48E**−**02)**
	5	9.80E−01 (1.77E−04)−	9.80E−01 (1.87E−04)−	9.80E−01 (1.44E−04)−	9.80E−01 (1.53E−04)−	**9.98E**−**01** **(1.89E**−**03)**
	8	9.88E−01 (4.43E−02)−	9.97E−01 (1.34E−04)−	9.98E−01 (5.52E−05)−	9.98E−01 (6.10E−05)−	**1.00E + 00** **(1.99E**−**04)**
	10	9.96E−01 (1.73E−02) =	**1.00E + 00** **(2.73E**−**05) = **	**1.00E + 00** **(1.56E**−**05) = **	**1.00E + 00** **(2.10E**−**05) = **	9.99E−01 (6.63E−04)
	15	9.87E−01 (1. 69E−02) =	1.00E + 00 (2.73E−04)−	**1.00E + 00** **(1.26E**−**05) + **	1.00E + 00 (5.47E−05) +	1.00E + 00 (2.07E−04)
DTLZ2	3	5.59E−01 (7.35E−05)−	5.59E−01 (6.76E−05)−	5.59E−01 (2.95E−04)−	5.59E−01 (1.84E−04)−	**9.26E**−**01** **(2.96E**−**03)**
	5	8.12E−01 (4.27E−04)−	8.12E−01 (4.57E−04)−	8.12E−01 (3.97E−04)−	8.12E−01 (3.82E−04)−	**9.90E**−**01** **(9.91E**−**04)**
	8	9.14E−01 (2.81E−02)−	9.24E−01 (2.69E−04)−	9.24E−01 (2.41E−04)−	9.25E−01 (4.83E−04)−	**9.99E**−**01** **(2.27E**−**03)**
	10	9.30E−01 (4.26E−02)−	9.70E−01 (1.85E−04)−	9.70E−01 (1.82E−04)−	9.71E−01 (1.94E−04)−	**1.00E + 00** **(1.05E**−**03)**
	15	8.55E−01 (6.01E−02)−	9.91E−01 (1.30E−04)−	9.90E−01 (2.21E−03)−	9.91E−01 (5.50E−04)−	**1.00E + 00** **(1.80E**−**05)**
DTLZ3	3	5.55E−01 (2.89E−03)−	5.54E−01 (4.35E−03)−	5.55E−01 (3.15E−03)−	5.55E−01 (3.39E−03)−	**8.81E**−**01** **(1.33E**−**01)**
	5	8.11E−01 (2.35E−03)−	8.10E−01 (1.88E−03)−	8.12E−01 (6.77E−04)−	8.11E−01 (1.77E−03)−	**9.89E**−**01** **(2.41E**−**03)**
	8	7.65E−01 (3.12E−01)−	8.22E−01 (2.30E−01)−	9.22E−01 (1.70E−03)−	9.26E−01 (2.85E−03)−	**9.96E**−**01** **(4.74E**−**03)**
	10	8.40E−01 (2.80E−01)−	9.70E−01 (6.71E−04)−	9.70E−01 (3.72E−04)−	9.71E−01 (3.71E−03)−	**1.00E + 00** **(4.97E**−**04)**
	15	0.00E + 00 (0.00E + 00)−	5.18E−01 (4.41E−01)−	9.91E−01 (1.33E−04)−	9.82E−01 (5.05E−03)−	**1.00E + 00** **(6.70E**−**05)**
DTLZ4	3	4.56E−01 (1.26E−01)−	4.13E−01 (1.65E−01)−	5.52E−01 (3.95E−02)−	4.64E−01 (1.38E−01)−	**8.76E**−**01** **(6.36E**−**02)**
	5	8.09E−01 (2.03E−02)−	7.43E−01 (8.12E−02)−	8.13E−01 (4.30E−04)−	8.09E−01 (1.69E−02)−	**9.80E**−**01** **(1.99E**−**02)**
	8	8.97E−01 (4.70E−02)−	8.68E−01 (4.18E−02)−	9.22E−01 (5.72E−03)−	9.25E−01 (3.41E−04)−	**9.97E**−**01** **(3.98E**−**03)**
	10	9.63E−01 (2.13E−02)−	9.43E−01 (2.42E−02)−	9.70E−01 (1.97E−04)−	9.70E−01 (1.84E−04)−	**1.00E + 00** **(2.08E**−**04)**
	15	8.97E−01 (4.70E−02)−	9.70E−01 (1.43E−02)−	9.90E−01 (1.48E−03)−	9.91E−01 (1.42E−04)−	**1.00E + 00** **(1.80E**−**05)**
DTLZ5	3	1.94E−01 (1.11E−03)−	1.82E−01 (1.05E−05)−	1.56E−01 (6.29E−03)−	1.99E−01 (1.16E−04)−	**7.26E**−**01** **(3.45E**−**03)**
	5	1.14E−01 (4.05E−03)−	1.27E−01 (3.49E−04)−	1.06E−01 (2.79E−03)−	1.14E−01 (1.83E−03)−	**6.55E**−**01** **(7.12E**−**03)**
	8	2.88E−02 (3.24E−02)−	1.04E−01 (3.84E−04)−	9.09E−02 (4.36E−05)−	9.17E−02 (2.41E−03)−	**6.23E**−**01** **(6.25E**−**03)**
	10	1.33E−02 (2.64E−02)−	1.00E−01 (3.87E−04)−	9.09E−02 (8.85E−05)−	8.86E−02 (1.67E−03)−	**6.24E**−**01** **(6.73E**−**03)**
	15	9.04E−02 (3.03E−03)−	9.44E−02 (2.27E−04)−	9.09E−02 (2.42E−04)−	9.12E−02 (5.54E−04)−	**6.01E**−**01** **(9.33E**−**03)**
DTLZ6	3	1.90E−01 (1.48E−03)−	1.82E−01 (1.26E−05)−	1.46E−01 (1.10E−02)−	2.00E−01 (6.13E−05)−	**7.18E**−**01** **(5.37E**−**03)**
	5	9.76E−02 (1.78E−02)−	1.27E−01 (1.78E−03)−	8.67E−02 (3.39E−02)−	1.09E−01 (5.81E−03)−	**6.61E**−**01** **(4.19E**−**03)**
	8	5.14E−02 (4.35E−02)−	1.04E−01 (2.93E−04)−	9.39E−02 (1.42E−03)−	9.24E−02 (1.37E−03)−	**6.19E**−**01** **(5.69E**−**03)**
	10	3.03E−03 (1.63E−02)−	9.99E−02 (2.35E−04)−	9.23E−02 (7.82E−04)−	9.18E−02 (7.95E−04)−	**6.14E**−**01** **(6.80E**−**03)**
	15	0.00E + 00 (0.00E + 00)−	9.44E−02 (3.15E−04)−	8.95E−02 (8.17E−03)−	9.15E−02 (6.17E−04)−	**5.88E**−**01** **(1.77E**−**02)**
DTLZ7	3	2.68E−01 (1.02E−02)−	2.47E−01 (6.21E−03)−	2.65E−01 (1.76E−03)−	2.49E−01 (2.38E−02)−	**4.10E**−**01** **(7.32E**−**02)**
	5	2.53E−01 (4.72E−03)−	1.47E−01 (1.26E−02)−	2.20E−01 (2.89E−03)−	2.54E−01 (1.81E−03)−	**4.41E**−**01** **(1.24E**−**02)**
	8	2.01E−01 (4.29E−03)−	4.17E−03 (1.52E−02)−	1.55E−01 (2.37E−02)−	1.87E−01 (2.88E−03)−	**3.47E**−**01** **(8.37E**−**03)**
	10	1.87E−01 (7.01E−03)−	7.97E−06 (2.37E−05)−	1.45E−01 (2.95E−02)−	1.67E−01 (8.07E−03)−	**3.13E**−**01** **(6.30E**−**03)**
	15	1.40E−01 (1.04E−02)−	2.46E−04 (1.27E−03)−	1.20E−01 (5.26E−03)−	7.16E−02 (1.62E−02)−	**1.67E**−**01** **(1.87E**−**02)**
+/=/−		0/2/33	0/1/34	1/1/33	1/1/33

**Table 7:** Friedman-test of 5 algorithms.

Algorithm	IGD		HV	
	Avg.rank	Rank	Avg.rank	Rank
NSGA-III	3.73	5	2.00	5
MOEA/D	2.97	3	2.34	4
RVEA	3.29	4	2.67	3
ARMOEA	2.79	2	3.11	2
MOIFF	2.23	1	4.87	1

Basing from the IGD results of DTLZ test instances shown in Table 5, we can find that the proposed MOIFF shows the best overall performance on DTLZ2 and DTLZ4 problems, compared with the four other MaOEAs. For the DTLZ1 problem, MOEA/D obtains the smallest IGD value on the fifteen-objective test instance, RVEA performs best on the three-objective test instance, while MOIFF works best on the five-, eight-, and ten-objective test instances. For DTLZ3, MOIFF has obvious advantages over the four other algorithms on the remaining test instances, and MOIFF is slightly worse than the four other algorithms on the three-objective test instance. For DTLZ5, NSGA-III achieves the best results on the three-objective test instance, whereas MOEA/D works best on the five-, eight-, ten-, and fifteen-objective test instances. The overall performance of MOIFF is significantly outperformed by NSGA-III and ARMOEA. For DTLZ6, the performance obtained by each algorithm is similar on all DTLZ5 instances. For DTLZ7, MOEA/D obtains the smallest IGD value on the fifteen-objective test instance, ARMOEA performs best on the five-objective test instance, and NSGA-III works best on the remaining test instances. According to the Table 7, the overall performance of MOIFF is significantly outperformed by the other competitors in terms of IGD is the best.

The HV results of DTLZ test instances are listed in Table 6. MOIFF can achieve the best results on all DTLZ instances except for ten- and fifteen-objective DTLZ instances. According to Table 7, we can see that, compared with the competitors, the overall performance of MOIFF over all test instances in terms of HV is the best.

(2) Verification of the effectiveness of MOIFF in convergence speed

To compare the complexity of each algorithm more intuitively, Table 8 records the random respond time of each algorithm on each test instance, where the parameters of each algorithm are set as above.

**Table 8:** The response time of the five algorithms on the DTLZ test suite with different numbers of objectives (/s).

Text Suite	*M*	NSGA-III	MOEA/D	RVEA	ARMOEA	MOIFF
DTLZ1	3	1.76	6.68	1.53	25.44	19.37
	5	9.15	27.40	8.13	545.94	130.42
	8	7.71	23.37	6.52	391.99	149.63
	10	21.16	60.25	14.22	1415.25	777.34
	15	18.25	41.07	12.98	808.41	299.35
DTLZ2	3	1.25	4.15	1.08	25.31	11.75
	5	6.19	16.32	4.76	375.87	63.92
	8	7.39	15.95	5.32	385.87	47.32
	10	16.19	50.63	13.39	1718.32	219.84
	15	16.10	27.61	8.45	647.24	310.61
DTLZ3	3	5.10	16.79	4.64	74.94	27.67
	5	15.99	46.65	14.12	699.76	136.36
	8	14.66	30.19	10.83	651.96	86.70
	10	40.22	101.73	28.01	2895.24	387.07
	15	30.07	54.81	22.56	1530.45	133.54
DTLZ4	3	5.05	4.79	4.37	141.00	17.57
	5	20.25	50.41	15.79	1342.95	138.55
	8	12.79	20.25	11.12	1322.76	106.81
	10	58.17	129.12	43.42	5216.51	516.42
	15	47.73	82.76	38.18	3000.08	265.89
DTLZ5	3	4.90	10.62	3.74	130.104	17.86
	5	19.03	47.09	11.53	1555.28	150.56
	8	18.65	38.83	12.17	1508.99	98.48
	10	18.33	40.56	10.48	1666.67	152.47
	15	33.11	56.77	25.17	2369.89	137.23
DTLZ6	3	3.68	7.23	2.77	13.85	11.15
	5	6.69	16.21	5.05	566.72	50.24
	8	12.98	24.55	9.04	980.66	44.76
	10	26.48	52.01	16.99	2361.61	205.54
	15	39.34	55.88	22.78	2420.85	134.48
DTLZ7	3	4.67	10.19	3.94	96.75	18.18
	5	18.62	45.16	12.74	1585.89	140.21
	8	19.51	40.86	13.60	1912.30	101.58
	10	62.77	127.04	36.07	6179.27	500.29
	15	53.54	79.87	32.27	4160.32	204.15

Seen from the Table 8, we can find that RVEA needs the shortest run time on each test instance, the respond time of ARMOEA is longest on each test instance, and MOIFF needs the longer run time than NSGA-III and MOEAD on each test instance.

**Figure 3:**
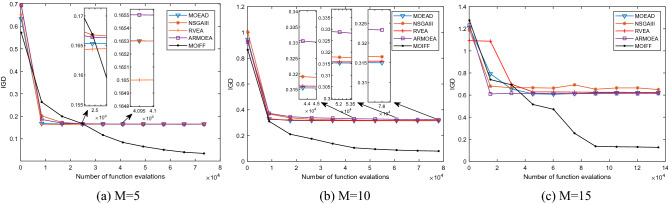
The convergence process curves of IGD values obtained by five algorithm on DTLZ2.

**Figure 4:**
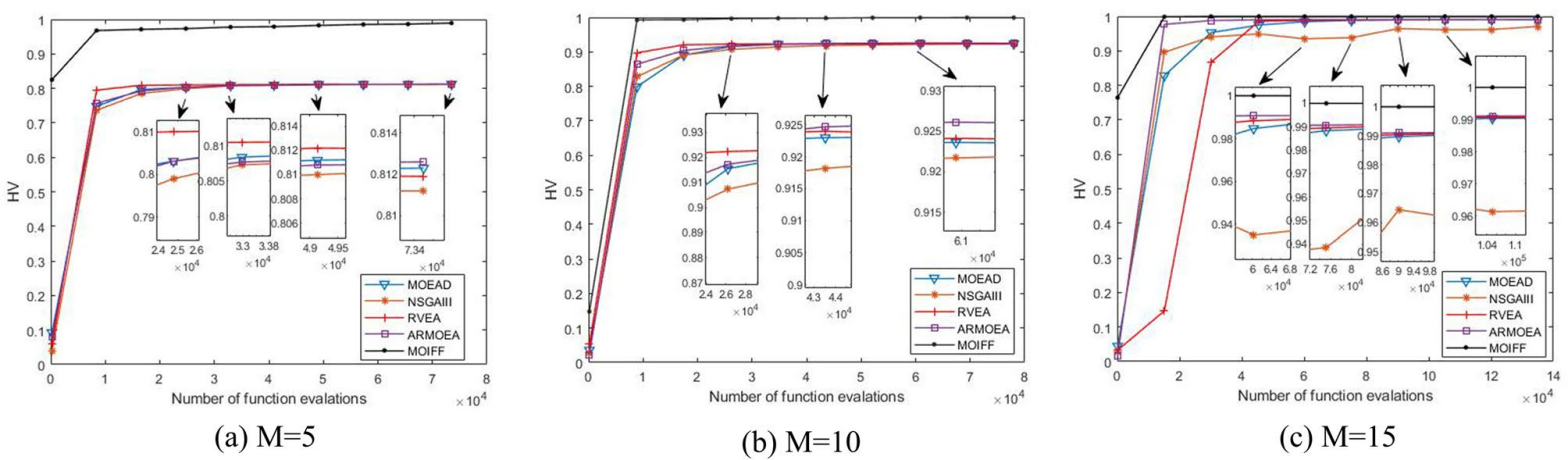
The convergence process curves of HV values obtained by five algorithm on DTLZ2.

**Figure 5:**
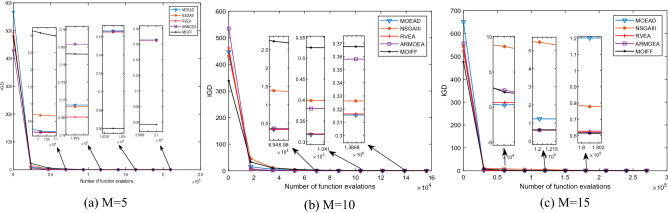
The convergence process curves of IGD values obtained by five algorithm on DTLZ3.

**Figure 6:**
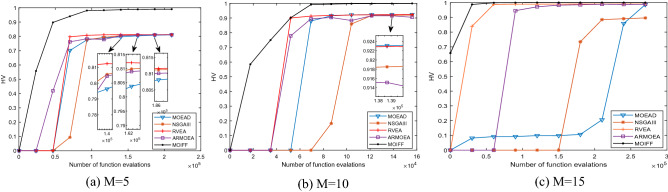
The convergence process curves of HV values obtained by five algorithm on DTLZ2.

In order to intuitively compare the convergence process of each algorithm, Figs. 3, 4, 5 and 6 shows the iterative process curves of each algorithm, where parameters of each algorithm are set as above. We can find that MOIFF is excellent in the convergence speed.

(3) Verification of the effectiveness of MOIFF in convergence stability

In order to compare the stability of each algorithm, we show the success rate of each algorithm in Table 9, where the success rate means the times of each algorithm reaching the preset IGD convergence accuracy in 30 independent experiments. Form Table 8, we can see that, MOIFF obtains the optimal success rate of 22 out of 35 test instances, which indicates that MOIFF is excellent in convergence stability.

**Table 9:** The success rate of the five algorithms on the DTLZ test suite with different numbers of objectives (/%).

Text suite	*M*	Convergence accuracy	NSGA-III	MOEA/D	RVEA	ARMOEA	MOIFF
DTLZ1	3	e−3	0	0	0	0	**70.00**
	5	e−3	0	0	0	0	**76.67**
	8	e−2	86.67	**100**	**100**	96.67	**100**
	10	e−2	0	0	0	0	**86.67**
	15	e−2	**100**	**100**	**100**	**100**	**100**
DTLZ2	3	e−2	**100**	**100**	**100**	**100**	**100**
	5	e−2	0	0	0	0	**100**
	8	e−2	0	0	0	0	**93.33**
	10	e−2	0	0	0	0	**10.00**
	15	e−2	0	0	0	0	**63.33**
DTLZ3	3	e−3	0	0	0	0	**6.67**
	5	e−2	0	0	0	0	**73.33**
	8	e−2	0	0	0	0	**20.00**
	10	e−2	0	0	0	0	**43.33**
	15	e−1	0	46.67	**100**	**100**	**100**
DTLZ4	3	e−3	0	0	0	0	**46.67**
	5	e−2	0	0	0	0	**76.67**
	8	e−2	0	0	0	0	**36.67**
	10	e−2	0	0	0	0	**70.00**
	15	e−2	0	0	0	0	**6.67**
DTLZ5	3	e−3	6.67	0	0	**100**	6.67
	5	e−2	73.33	**100**	**0**	**100**	80.00
	8	e−2	0	**100**	0	46.67	13.33
	10	e−2	0	**100**	0	53.55	10.00
	15	e−2	0	**100**	0	33.33	3.33
DTLZ6	3	e−3	0	0	0	**100**	0
	5	e−2	23.33	**100**	0	86.67	93.33
	8	e−2	0	**100**	0	50.00	0
	10	e−2	0	**100**	0	53.33	0
	15	e−2	0	**100**	0	50.00	0
DTLZ7	3	e−2	**93.33**	0	0	36.67	13.33
	5	e−1	**100**	**100**	**100**	**100**	**100**
	8	e−1	**100**	0	0	50.00	43.33
	10	e−1	**66.67**	0	0	30.00	0
	15	e + 0	**100**	**100**	**100**	**100**	**100**

**Figure 7:**
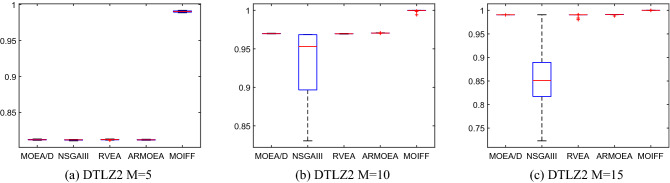
The box plots of HV values obtained by five algorithm on DTLZ2 over 30 runs.

**Figure 8:**
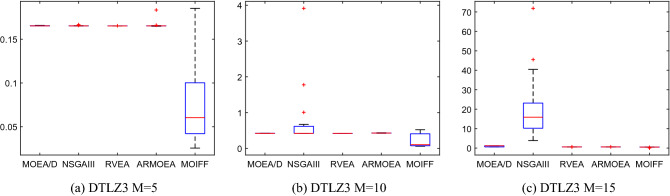
The box plots of IGD values obtained by five algorithm on DTLZ3 over 30 runs.

To further visually compare the stability of each algorithm, Figs. 7 and 8 show the box plots of statistical values of IGD and HV obtained by the above five algorithms over 30 runs. Limited to the length of the paper, we only select the IGD values obtained by the five algorithms on the DTLZ2 and the HV values obtained by the five algorithms on the DTLZ3 to draw the box plots. We can see that MOIFF is the most stable compare to the other 4 algorithms for HV. For five- and ten-objective DTLZ3, the stability of IGD values obtained by IFF is slightly inferior to the other algorithms, but its IGD value is significantly best.

### Results on WFG test suite

Comparison results of MOIFF with the four other MaOEAs in terms of IGD values on the WFG test suite are listed in Table 10. MOIFF is significantly outperformed by the four other MOEAs on the WFG4, WFG5, WFG6, WFG7, WFG8 and WFG9 instances in terms of IGD. For WFG1, RVEA obtains the smallest IGD value on the fifteen-objective test instance, ARMOEA performs best on the eight-objective test instance, and MOIFF works best on the remaining test instances. For WFG2, RVEA performs best on the five-objective test instance, and MOIFF works best on the remaining test instances. For WFG3, except for the three-objective instance, MOIFF can achieve the best performance on the other test instances. MOEA/D could obtain the best results on any test instance.

**Table 10:** Average and standard deviation of the IGD values obtained by the five algorithms on the WFG test suite with different numbers of objectives.

Text suite	M	NSGA-III	MOEA/D	RVEA	ARMOEA	MOIFF
WFG1	3	**1.48E**−**01 (3.61E**−**03) + **	2.42E−01 (7.46E−03)−	1.74E−01 (1.24E−02) +	1.48E−01 (2.84E−03) +	2.29E−01 (2.92E−02)
	5	**3.63E**−**01** **(3.59E**−**03) + **	7.03E−01 (1.50E−02) +	3.67E−01 (4.56E−03) +	3.70E−01 (3.14E−03) +	7.78E−01 (1.52E−01)
	8	8.78E−01 (2.33E−02) +	1.48E + 00 (5.59E−02) +	9.79E−−01 (3.64E−02) +	**8.72E**−**01** **(1.89E**−**02) + **	2.76E + 00 (5.26E−01)
	10	**9.54E**−**01** **(1.62E**−**02) + **	1.69E + 00 (6.97E−02) +	1.06E + 00 (2.41E−02) +	1.02E + 00 (2.85E−02) +	2.61E + 00 (4.60E−01)
	15	2.14E + 00 (4.13E−01) +	2.96E + 00 (2.43E−01) +	**1.77E + 00** **(8.15E**−**02) + **	1.80E + 00 (4.60E−02) +	6.86E + 00 (8.41E−01)
WFG2	3	1.64E−01 (9.43E−04) +	2.36E−01 (1.52E−02) =	1.81E−01 (4.47E−03) +	**1.64E**−**01** **(1.13E**−**03) + **	2.45E−01 (1.37E−02)
	5	3.91E−01 (1.57E−03) +	7.82E−01 (1.35E−01)−	**3.81E**−**01** **(6.84E**−**03) + **	3.92E−01 (1.98E−03) +	5.90E−01 (2.75E−02)
	8	1.03E + 00 (2.03E−01) +	1.70E + 00 (1.78E−02)−	9.89E−01 (3.46E−02) +	**9.44E**−**01** **(9.27E**−**03) + **	1.27E + 00 (8.74E−02)
	10	1.33E + 00 (1.86E−01) +	1.83E + 00 (1.24E−02)−	1.06E + 00 (3.26E−02) +	**1.01E + 00** **(1.59E**−**02) + **	1.50E + 00 (8.08E−02)
	15	2.13E + 00 (1.39E−01) +	2.55E + 00 (1.84E−02) +	1.87E + 00 (6.72E−02) +	**1.72E + 00** **(3.47E**−**02) + **	3.66E + 00 (2.36E−01)
WFG3	3	**1.02E**−**01** **(1.02E**−**02) + **	1.57E−01 (1.12E−03) =	2.22E−01 (1.23E−02)−	1.15E−01 (7.74E−03) +	1.53E−01 (1.45E−02)
	5	4.37E−01 (4.08E−02)−	8.03E−01 (3.75E−01)−	4.38E−01 (9.30E−03)−	4.49E−01 (3.56E−02)−	**2.75E**−**01** **(5.93E**−**02)**
	8	1.28E + 00 (5.07E−01)−	3.62E + 00 (1.69E−01)−	2.11E + 00 (2.57E−01)−	1.81E + 00 (1.12E−01)−	**7.93E**−**01** **(1.74E**−**01)**
	10	1.94E + 00 (4.25E−01)−	1.83E + 00 (1.24E−02)−	3.08E + 00 (3.85E−01)−	2.45E + 00 (8.92E−02)−	**7.86E**−**01** **(1.12E**−**01)**
	15	3.73E + 00 (1.54E + 00)−	9.15E + 00 (2.29E−01)−	6.82E + 00 (1.33E + 00)−	5.59E + 00 (9.67E−02)−	**1.82E + 00** **(2.84E**−**01)**
WFG4	3	2.21E−01 (7.18E−05)−	2.54E−01 (4.08E−03)−	2.28E−01 (3.34E−03)−	2.21E−01 (9.66E−05)−	**5.63E**−**02** **(1.39E**−**02)**
	5	9.68E−01 (4.56E−04)−	1.57E + 00 (3.65E−01)−	9.67E−01 (5.65E−04)−	9.67E−01 (3.79E−04)−	**1.41E**−**01** **(1.58E**−**02)**
	8	2.99E + 00 (1.04E−01)−	6.47E + 00 (3.36E−01)−	2.97E + 00 (9.52E−03)−	2.96E + 00 (3.20E−03)−	**6.56E**−**01** **(3.35E**−**01)**
	10	4.55E + 00 (1.77E−02)−	8.44E + 00 (4.12E−01)−	4.45E + 00 (2.65E−02)−	4.55E + 00 (9.26E−03)−	**8.11E**−**01** **(2.18E**−**02)**
	15	9.65E + 00 (3.97E−01)−	1.56E + 01 (2.72E−01)−	9.21E + 00 (9.97E−02)−	9.38E + 00 (3.19E−02)−	**1.78E + 00** **(1.38E + 00)**
WFG5	3	2.30E−01 (3.88E−05)−	2.48E−01 (2.37E−03)−	2.31E−01 (3.77E−04)−	2.30E−01 (4.74E−05)−	**1.37E**−**01** **(3.75E**−**02)**
	5	9.59E−01 (1.98E−04)−	1.48E + 00 (3.59E−01)−	9.59E−01 (1.92E−04)−	9.59E−01 (1.68E−04)−	**1.88E**−**01** **(3.14E**−**02)**
	8	2.94E + 00 (1.39E−03)−	6.02E + 00 (2.11E−01)−	2.95E + 00 (8.43E−03)−	2.94E + 00 (1.99E−03)−	**5.93E**−**01** **(6.54E**−**02)**
	10	4.53E + 00 (3.33E−03)−	7.92E + 00 (2.76E−01)−	4.42E + 00 (2.52E−02)−	4.53E + 00 (7.72E−03)−	**8.30E**−**01** **(4.33E**−**02)**
	15	9.31E + 00 (1.39E−01)−	1.51E + 01 (1.21E−01)−	9.16E + 00 (5.63E−02)−	9.34E + 00 (5.68E−02)−	**3.02E + 00** **(1.51E + 00)**
WFG6	3	2.34E−01 (9.47E−03)−	2.78E−01 (1.52E−02)−	2.46E−01 (1.17E−02)−	2.40E−01 (8.94E−03)−	**1.51E**−**01** **(4.70E**−**02)**
	5	9.60E−01 (7.65E−04)−	1.57E + 00 (3.13E−01)−	9.60E−01 (1.12E−03)−	9.59E−01 (6.72E−04)−	**2.32E**−**01** **(3.50E**−**02)**
	8	2.95E + 00 (4.20E−03)−	6.93E + 00 (7.53E−02)−	2.97E + 00 (1.12E−02)−	2.95E + 00 (3.35E−03)−	**9.24E**−**01** **(3.34E**−**01)**
	10	4.64E + 00 (4.87E−01)−	9.16E + 00 (1.43E−01)−	4.39E + 00 (2.96E−02)−	4.53E + 00 (9.26E−03)−	**8.95E**−**01** **(2.92E**−**02)**
	15	1.18E + 01 (8.86E−01)−	1.61E + 01 (2.05E−01)−	9.50E + 00 (3.69E−01)−	9.41E + 00 (3.57E−02)−	**3.10E + 00** **(1.30E + 00)**
WFG7	3	2.21E−01 (4.27E−04)−	2.91E−01 (1.76E−02)−	2.24E−01 (1.58E−03)−	2.21E−01 (1.48E−04)−	**2.67E**−**02** **(9.61E**−**03)**
	5	9.68E−01 (3.81E−04)−	1.69E + 00 (1.49E−01)−	9.69E−01 (4.68E−04)−	9.69E−01 (6.97E−04)−	**8.72E**−**02** **(1.52E**−**02)**
	8	2.97E + 00 (5.59E−03)−	7.00E + 00 (1.03E−01)−	2.99E + 00 (1.68E−02)−	2.96E + 00 (4.89E−03)−	**7.23E**−**01** **(2.39E**−**02)**
	10	4.61E + 00 (2.55E−01)−	9.08E + 00 (2.58E−01)−	4.45E + 00 (2.84E−02)−	4.52E + 00 (1.94E−02)−	**8.64E**−**01** **(1.56E**−**02)**
	15	9.44E + 00 (3.25E−01)−	1.63E + 01 (8.25E−02)−	9.33E + 00 (6.49E−02)−	9.39E + 00 (4.26E−02)−	**3.16E + 00** **(7.08E**−**01)**
WFG8	3	2.82E−01 (2.57E−03)−	3.06E−01 (5.35E−03)−	2.92E−01 (3.19E−03)−	2.73E−01 (1.79E−03) =	**2.69E**−**01** **(1.20E**−**02)**
	5	9.95E−01 (6.40E−03)−	1.33E + 00 (2.75E−01)−	9.86E−01 (8.62E−04)−	9.81E−01 (1.35E−03)−	**6.05E**−**01** **(4.21E**−**02)**
	8	3.52E + 00 (4.10E−01)−	6.32E + 00 (2.43E−01)−	3.05E + 00 (2.54E−02)−	3.02E + 00 (3.03E−02)−	**2.16E + 00** **(4.80E**−**01)**
	10	4.94E + 00 (3.78E−01)−	8.62E + 00 (2.94E−01)−	4.40E + 00 (6.03E−02)−	4.63E + 00 (4.38E−02)−	**3.08E + 00** **(8.23E**−**01)**
	15	1.11E + 01 (7.78E−01)−	1.46E + 01 (1.35E + 00)−	9.37E + 00 (4.82E−01)−	9.39E + 00 (4.23E−02)−	**6.16E + 00** **(7.03E**−**01)**
WFG9	3	2.23E−01 (1.12E−03)−	2.68E−01 (2.91E−02)−	2.24E−01 (2.05E−03)−	2.25E−01 (2.17E−02)−	**9.18E**−**02** **(5.04E**−**02)**
	5	9.39E−01 (3.29E−03)−	1.39E + 00 (1.41E−01)−	9.48E−01 (1.26E−03)−	9.47E−01 (2.11E−03)−	**3.23E**−**01** **(3.64E**−**02)**
	8	2.93E + 00 (1.01E−02)−	6.41E + 00 (1.62E−01)−	2.95E + 00 (1.89E−02)−	2.93E + 00 (5.57E−03)−	**1.03E + 00** **(1.27E**−**01)**
	10	4.47E + 00 (1.23E−01)−	8.61E + 00 (6.55E−01)−	4.32E + 00 (3.44E−02)−	4.50E + 00 (1.38E−02)−	**1.58E + 00** **(4.21E**−**01)**
	15	9.11E + 00 (3.59E−01)−	1.46E + 01 (2.03E + 00)−	9.17E + 00 (8.39E−02)−	9.12E + 00 (3.88E−02)−	**4.36E + 00** **(3.85E**−**01)**
+/=/−		11/0/34	5/2/38	10/0/35	11/1/33	

The HV results of the WFG test instances are listed in Table 11. NSGA-III can obtain the best HV values on the three-, five-, and fifteen-objective WFG1 instances; the five-objective WFG2 instance; and the eight-objective WFG3 instance. MOEA/D only works best on the ten-objective WFG3 instance. RVEA performs best on the fifteen-objective WFG5 instance, and the ten- and fifteen-objective WFG9 instances. ARMOEA works best on the eight- and ten-objective WFG1 instances, eight-, ten-, and fifteen-objective WFG2 instances, fifteen-objective WFG4 instance; the fifteen-objective WFG6 instance; the fifteen-objective WFG7 instance; and the eight-, ten-, and fifteen-objective WFG8 instances. Among the 45 test instances, MOIFF obtains the smallest HV values on 24 test instances.

**Table 11:** Average and standard deviation of the HV values obtained by the five algorithms on the WFG test suite with different numbers of objectives.

Text suite	M	NSGAIII	MOEA/D	RVEA	ARMOEA	MOIFF
WFG1	3	**9.43E**−**01** **(4.52E**−**03) + **	9.01E−01 (1.44E−02)−	9.38E−01 (2.74E−03) =	9.43E−01 (7.00E−04) +	9.37E−01 (1.52E−02)
	5	**9.98E**−**01** **(1.44E**−**04) + **	9.49E−01 (1.58E−02)−	9.98E−01 (1.22E−04) +	9.94E−01 (5.00E−03) +	9.72E−01 (1.36E−02)
	8	9.99E−01 (3.14E−04) +	8.27E−01 (6.78E−02) =	9.96E−01 (3.75E−03) +	**1.00E + 00** **(4.23E**−**05) + **	8.66E−01 (6.33E−02)
	10	9.99E−01 (2.64E−04) +	7.35E−01 (1.05E−01)−	9.97E−01 (5.13E−04) +	**1.00E + 00** **(2.67E**−**04) + **	9.29E−01 (3.31E−02)
	15	**9.99E**−**01** **(7.15E**−**04) + **	4.30E−01 (9.42E−02)−	9.86E−01 (3.57E−02) +	9.99E−01 (5.81E−04) +	8.48E−01 (7.10E−02)
WFG2	3	9.31E−01 (5.84E−04)−	9.14E−01 (5.26E−03)−	9.28E−01 (8.48E−04)−	9.31E−01 (8.75E−04)−	**9.40E**−**01** **(3.66E**−**03)**
	5	**9.97E**−**01** **(4.43E**−**04) + **	9.33E−01 (5.96E−02)−	9.96E−01 (4.76E−04) +	9.92E−01 (5.01E−03) +	9.76E−01 (3.09E−03)
	8	9.97E−01 (1.18E−03) +	9.37E−01 (4.13E−03)−	9.85E−01 (4.88E−03)−	**9.98E**−**01** **(8.24E**−**04) + **	9.92E−01 (2.62E−03)
	10	9.98E−01 (1.04E−03) +	9.41E−01 (2.92E−03)−	9.89E−01 (2.61E−03)−	**9.98E**−**01** **(6.76E**−**04) + **	9.96E−01 (1.15E−03)
	15	9.94E−01 (6.55E−03) +	9.26E−01 (7.65E−03)−	9.73E−01 (6.91E−03)−	**9.97E**−**01** **(1.47E**−**03) + **	9.92E−01 (2.37E−03)
WFG3	3	**3.90E**−**01** **(3.83E**−**03)**−	3.65E−01 (7.90E−04)−	3.41E−01 (6.28E−03)−	3.81E−01 (3.31E−03)−	5.57E−01 (6.94E−03)
	5	1.77E−01 (1.21E−02)−	7.31E−02 (5.56E−02)−	1.79E−01 (7.41E−03)−	1.66E−01 (1.18E−02)−	**3.98E**−**01** **(1.56E**−**02)**
	8	**6.35E**−**02** **(2.08E**−**02)**−	0.00E + 00 (0.00E + 00)−	0.00E + 00 (0.00E + 00)−	2.61E−02 (2.40E−02)−	2.54E−01 (1.39E−02)
	10	6.57E−03 (1.12E−02)−	**9.41E**−**01** **(2.92E**−**03) + **	0.00E + 00 (0.00E + 00)−	0.00E + 00 (0.00E + 00)−	2.19E−01 (1.43E−02)
	15	0.00E + 00 (0.00E + 00)−	0.00E + 00 (0.00E + 00)−	0.00E + 00 (0.00E + 00)−	0.00E + 00 (0.00E + 00)−	**1.28E**−**01** **(1.52E**−**02)**
WFG4	3	5.59E−01 (2.59E−04)−	5.44E−01 (9.99E−04)−	5.55E−01 (1.09E−03)−	5.59E−01 (1.78E−04)−	**7.30E**−**01** **(4.41E**−**03)**
	5	8.09E−01 (8.55E−04)−	6.77E−01 (1.07E−01)−	8.08E−01 (8.38E−04)−	8.04E−01 (5.03E−03)−	**8.88E**−**01** **(3.03E**−**03)**
	8	9.11E−01 (1.85E−02)−	4.88E−01 (4.38E−02)−	9.15E−01 (1.98E−03)−	9.17E−01 (1.40E−03)−	**9.50E**−**01** **(8.53E**−**03)**
	10	9.59E−01 (6.08E−03)−	4.92E−01 (4.43E−02)−	9.60E−01 (1.82E−03)−	9.61E−01 (1.14E−03)−	**9.80E**−**01** **(1.03E**−**03)**
	15	8.82E−01 (3.46E−02)−	3.35E−01 (4.29E−02)−	9.80E−01 (3.05E−03) =	**9.85E**−**01** **(1.67E**−**03) = **	9.73E−01 (2.19E−02)
WFG5	3	5.18E−01 (2.70E−05)−	5.03E−01 (5.07E−03)−	5.17E−01 (2.48E−04)−	5.18E−01 (8.94E−05)−	**6.93E**−**01** **(6.47E**−**03)**
	5	7.61E−01 (3.85E−04)−	6.55E−01 (8.20E−02)−	7.61E−01 (3.34E−04)−	7.57E−01 (5.02E−03)−	**8.45E**−**01** **(3.40E**−**03)**
	8	8.63E−01 (3.14E−04)−	5.29E−01 (1.47E−02)−	8.62E−01 (4.47E−04)−	8.61E−01 (1.07E−03)−	**8.96E**−**01** **(2.10E**−**03)**
	10	9.04E−01 (3.42E−04)−	5.48E−01 (1.61E−02)−	9.03E−01 (3.76E−04)−	9.01E−01 (4.27E−03)−	**9.21E**−**01** **(1.36E**−**03)**
	15	9.00E−01 (3.37E−02) +	3.43E−01 (5.63E−02)−	**9.17E**−**01** **(1.74E**−**04) + **	9.15E−01 (5.49E−04) +	8.76E−01 (3.01E−02)
WFG6	3	5.14E−01 (1.47E−02)−	4.90E−01 (1.79E−02)−	5.02E−01 (1.56E−02)−	5.05E−01 (1.34E−02)−	**6.81E**−**01** **(1.78E**−**02)**
	5	7.46E−01 (9.83E−03)−	6.08E−01 (7.28E−02)−	7.45E−01 (1.77E−02)−	7.38E−01 (1.08E−02)−	**8.23E**−**01** **(1.54E**−**02)**
	8	8.52E−01 (1.23E−02)−	2.89E−01 (3.17E−02)−	8.44E−01 (1.90E−02)−	8.40E−01 (1.83E−02)−	**8.66E**−**01** **(2.77E**−**02)**
	10	8.72E−01 (2.58E−02)−	2.86E−01 (2.41E−02)−	8.76E−01 (1.78E−02)−	8.72E−01 (1.52E−02)−	**8.97E**−**01** **(2.00E**−**02)**
	15	6.95E−01 (4.64E−02)−	1.18E−01 (3.33E−02)−	7.53E−01 (6.15E−02)−	**8.87E**−**01** **(2.06E**−**02) + **	8.63E−01 (4.28E−02)
WFG7	3	5.58E−01 (1.91E−04)−	5.32E−01 (3.68E−03)−	5.55E−01 (7.58E−04)−	5.58E−01 (2.24E−04)−	**7.31E**−**01** **(3.05E**−**03)**
	5	8.10E−01 (5.15E−04)−	6.63E−01 (4.89E−02)−	8.08E−01 (5.19E−04)−	8.02E−01 (5.05E−03)−	**8.88E**−**01** **(2.54E**−**03)**
	8	9.19E−01 (1.30E−03)−	3.64E−01 (2.01E−02)−	9.09E−01 (2.60E−03)−	9.16E−01 (1.49E−03)−	**9.50E**−**01** **(1.89E**−**03)**
	10	9.63E−01 (1.06E−02)−	3.67E−01 (1.45E−02)−	9.58E−01 (1.99E−03)−	9.58E−01 (4.98E−03)−	**9.75E**−**01** **(1.37E**−**03)**
	15	9.62E−01 (2.92E−02) +	1.60E−01 (8.80E−03)−	9.63E−01 (2.58E−02) +	**9.85E**−**01** **(1.95E**−**03) + **	9.38E−01 (1.30E−02)
WFG8	3	4.73E−01 (1.91E−03)−	4.64E−01 (2.33E−03)−	4.72E−01 (1.40E−03)−	4.78E−01 (1.18E−03)−	**6.51E**−**01** **(5.82E**−**03)**
	5	6.98E−01 (2.17E−03)−	5.93E−01 (1.05E−01)−	7.02E−01 (1.25E−03)−	7.02E−01 (5.10E−03)−	**7.66E**−**01** **(3.83E**−**03)**
	8	7.62E−01 (3.98E−02) =	8.94E−02 (5.32E−02)−	7.65E−01 (5.85E−02) =	**8.25E**−**01** **(2.92E**−**02) + **	7.73E−01 (2.28E−02)
	10	8.34E−01 (3.15E−02) =	7.90E−02 (4.62E−02)−	7.99E−01 (7.39E−02)−	**9.21E**−**01** **(1.97E**−**02) + **	8.38E−01 (2.46E−02)
	15	5.63E−01 (9.70E−02)−	1.11E−01 (2.31E−01)−	6.22E−01 (1.37E−01)−	**9.45E**−**01** **(2.64E**−**02) + **	8.29E−01 (7.94E−03)
WFG9	3	5.36E−01 (2.32E−03)−	4.96E−01 (2.61E−02)−	5.38E−01 (1.66E−03)−	5.32E−01 (2.28E−02)−	**7.07E**−**01** **(1.91E**−**02)**
	5	7.67E−01 (4.04E−03)−	6.10E−01 (1.00E−01)−	7.72E−01 (2.88E−03)−	7.50E−01 (8.16E−03)−	**8.47E**−**01** **(6.35E**−**03)**
	8	8.23E−01 (5.90E−02)−	4.14E−01 (7.76E−02)−	8.48E−01 (1.63E−02)−	8.20E−01 (2.52E−02)−	**8.57E**−**01** **(4.22E**−**02)**
	10	8.70E−01 (5.53E−02) =	3.66E−01 (1.40E−01)−	**8.88E**−**01** **(3.27E**−**02) = **	8.45E−01 (3.61E−02)−	8.75E−01 (4.46E−02)
	15	8.07E−01 (6.83E−02) =	2.04E−01 (1.67E−01)−	**8.36E**−**01** **(5.40E**−**02) + **	8.18E−01 (4.20E−02) =	7.99E−01 (5.41E−02)
+/=/−		11/4/30	1/1/43	7/4/34	15/2/18	

**Figure 9:**
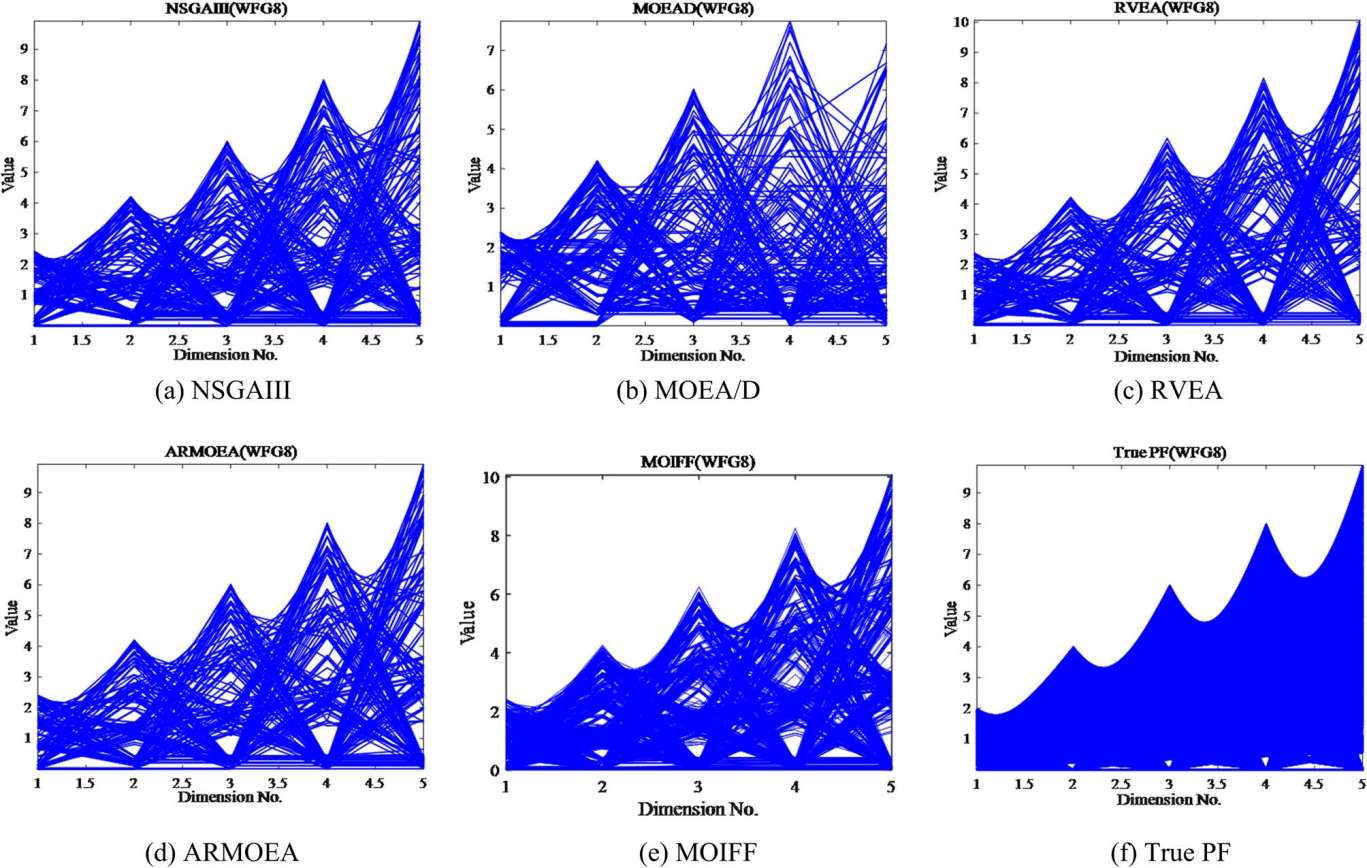
Parallel coordinates of final non-dominated solutions obtained by five algorithms on the five-objective WFG8 test suite.

Figure 9 shows the parallel coordinates of the final non-dominated solutions obtained by these five algorithms on the five-objective WFG8 test instance. These plots clearly demonstrate that the PF obtained by MOIFF is close to the true PF and maintains a good distribution.

## Conclusions

A novel algorithm named MOIFF is proposed in this paper for handling MaOPs to improve the comprehensive performance in terms of convergence and diversity. FF with excellent convergence performance is employed to serve as the optimization strategy of MOIFF. In order to handle MaOPs effectively, FF has been tailored from the following aspects in this paper. First, to distinguish the quality of each individual in MaOPs, we propose a novel individual fitness assessment approach based on cumulative ranking value. Second, considering the characteristics of MaOPs, we propose a novel method based on individual cumulative ranking value to constitute and update the global memory and local memory of each individual and a hybrid search mode combining subspace search and full space search to update individuals at the stages of soil optimization and soil fusion. In addition, we improve the dual aggregation function-based environmental selection. Finally, the results on the DTLZ and WFG test suites show that MOIFF has excellent convergence and diversity compared with four state-of-the art many-objective evolutionary algorithms.

## Data Availability

The data that support the findings of this study are available from the corresponding author upon reasonable request.
